# A Multimodal Sensing CMOS Imager Based on Dual‐Focus Imaging

**DOI:** 10.1002/advs.202206699

**Published:** 2023-03-02

**Authors:** Hao Dong, Xubin Zheng, Chen Cheng, Libin Qian, Yaoxuan Cui, Weiwei Wu, Qingjun Liu, Xing Chen, Yanli Lu, Qing Yang, Fenni Zhang, Di Wang

**Affiliations:** ^1^ Intelligent Perception Research Institute Zhejiang Lab Hangzhou 311100 China; ^2^ School of Advanced Materials and Nanotechnology Interdisciplinary Research Center of Smart Sensors Xidian University Shaanxi 710126 China; ^3^ Biosensor National Special Laboratory Key Laboratory for Biomedical Engineering of Education Ministry College of Biomedical Engineering and Instrument Science Zhejiang University Hangzhou 310027 China; ^4^ State Key Laboratory of Modern Optical Instrumentation College of Optical Science and Engineering Zhejiang University Joint International Research Laboratory of Photonics Hangzhou 310027 China

**Keywords:** CMOS imagers, colorimetric arrays, lab on chip, lensless imaging, multimodal sensors

## Abstract

Advanced machine intelligence is empowered not only by the ever‐increasing computational capability for information processing but also by sensors for collecting multimodal information from complex environments. However, simply assembling different sensors can result in bulky systems and complex data processing. Herein, it is shown that a complementary metal‐oxide‐semiconductor (CMOS) imager can be transformed into a compact multimodal sensing platform through dual‐focus imaging. By combining lens‐based and lensless imaging, visual information, chemicals, temperature, and humidity can be detected with the same chip and output as a single image. As a proof of concept, the sensor is equipped on a micro‐vehicle, and multimodal environmental sensing and mapping is demonstrated. A multimodal endoscope is also developed, and simultaneous imaging and chemical profiling along a porcine digestive tract is achieved. The multimodal CMOS imager is compact, versatile, and extensible and can be widely applied in microrobots, in vivo medical apparatuses, and other microdevices.

## Graphical abstract

This work transforms a complementary metal‐oxide‐semiconductor imager into a multimodal sensing platform (M‐imager) through a dual‐focus imaging strategy, and a fingernail‐sized M‐imager can detect visual information, chemicals, humidity, and temperature. The M‐imager is compact and versatile, which is anticipated to find wide applications in smart microdevices.

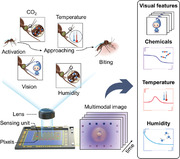

## 1 Introduction

Synergistic perception of multimodal information is a fundamental skill developed by animals through long evolutionary processes, which is crucial for survival and competition in complex environments.^[^ ^1^ ^]^ For instance, mosquitoes detect odors, temperature, humidity, and visual features to locate and recognize potential hosts.^[^ ^2^ ^]^ Carnivores exploit olfactory and visual information to hunt prey.^[^ ^3^ ^]^ Human beings grasp objects by sight and touch, and judge the freshness of food by sight and smell. Inspired by natural intelligence, various multimodal sensing systems have been proposed for different applications, achieving notable progress. For example, stretchable strain sensors and image sensors have been used to collect somatosensory and visual data for accurate and intelligent gesture recognition.^[^ ^4^ ^]^ Olfactory and tactile sensor arrays have been integrated in mechanical hands to mimic the sense‐fusion system of the star‐nose mole, and this system has demonstrated robust object recognition in nonvisual environments.^[^ ^5^ ^]^ The combination of electronic nose and machine vision has also been explored for food and beverage classification.^[^ ^6^ ^]^

Despite some progress, existing multimodal sensing technologies assemble various sensors based on different sensing principles, resulting in bulky system designs, cumbersome signal interfaces, and complex data processing procedures.^[^ ^7^ ^]^ These technologies cannot meet the growing complex environment perception demands^[^ ^8^ ^]^ of miniaturized devices, such as microrobots^[^ ^9^ ^]^ and in vivo medical devices.^[^ ^10^ ^]^ Thus, a miniature multimodal sensing platform is urgently needed. For most animal and biomimetic systems, vision is an indispensable sensory input that collects the majority of information.^[^ ^11^ ^]^ Thus, integrating other sensing modalities with vision sensors is the preferred approach for developing multimodal sensors.

As the most commonly used vision sensor, the small complementary metal‐oxide‐semiconductor (CMOS) imager chip offers millions of pixels, and each pixel serves as a photoelectric transducer with a high signal‐to‐noise ratio, which provides considerable potential for the modification and integration of other sensing modalities.^[^ ^12^ ^]^ For example, compound microlenses with different focal lengths have been 3D printed on CMOS imagers to simulate the foveated imaging of eagle eyes.^[^ ^13^ ^]^ A miniaturized spectrometer with a high spectral resolution was created by the complex optical interference achieved by positioning photonic crystal slabs on top of CMOS imager pixels.^[^ ^14^ ^]^ In addition to visual applications, CMOS imagers have been applied as gas sensors by printing microdroplets containing chemically sensitive dyes directly on the pixels.^[^ ^15^ ^]^ These works have demonstrated the versatility of the CMOS imager as a sensing platform; however, each work focused on only one sensing modality.

Herein, we present a multimodal sensing CMOS imager (M‐imager) based on a dual‐focus imaging strategy that combines traditional lens‐based imaging for visual information acquisition with lensless imaging to integrate other sensing modalities.^[^ ^16^ ^]^ Mimicking the multimodal information collection of mosquitoes during host location, we printed colorimetric sensing materials on the imager surface to detect carbon dioxide (CO_2_), temperature, and humidity. These sensing units and visual information can be captured in a single image, which enables extracting more effective characteristics without dimensional correlation and facilitates further data processing and analysis. To demonstrate the multimodal sensing capability of the M‐imager in real application scenarios, we equipped a micro remote‐controlled vehicle with the M‐imager and evaluated its performance in detecting and mapping multimodal information in different environments. We also developed a multimodal endoscope and demonstrated simultaneous imaging and gas profiling along a porcine digestive tract.

## 2 Results

### 2.1 Principle of the Multimodal Sensing CMOS Imager

**Figure 1:**
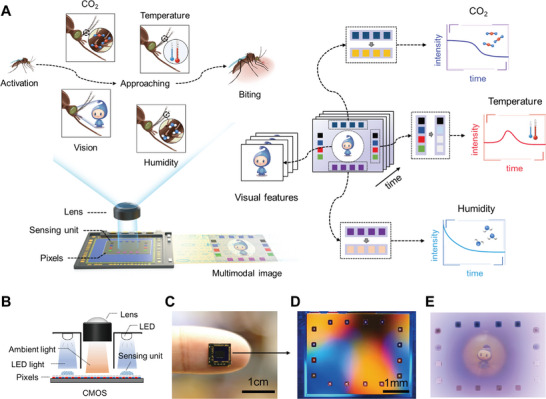
Principle of the multimodal sensing CMOS imager (M‐imager). A) Schematic illustration of an M‐imager that can detect multiple parameters that are involved in the host location of mosquitoes. B) Schematic diagram of the combination of lens‐based and lensless imaging on a single CMOS imager chip. C) Photograph of the fingernail‐sized M‐imager chip. D) Partial close‐up of the M‐imager's imaging region with multiple sensing units in (C). E) A typical multimodal image captured by the M‐imager.

We transformed a conventional CMOS imager into the proposed M‐imager using a dual‐focus imaging strategy in which lens‐based imaging and lensless imaging were combined on a single imager chip. As shown in **Figure** **1**A,B, the central region of the imaging area worked in the conventional lens‐based imaging mode to acquire visual information. To integrate other sensing modalities, micro colorimetric sensing units were directly coated on the edge region in the imaging area (Figure 1D), and their color responses were clearly captured without introducing additional optical components due to the zero focal distance and pixel size resolution of the lensless imaging mode (Figure 1E). The partitions in the sensor housing eliminate interference from ambient light on the lensless imaging area. To mimic the perceptual capability of mosquitoes in host recognition and localization, we coated the nail‐sized imager chip with different colorimetric units to detect CO_2_, temperature, and humidity (Figure 1C,D). The temperature sensing units contain thermochromic microcapsules composed of lyochromic dyes, color agents, and solvents. At low temperature, the solvent solidifies, and the color agent changes the structure of the lyochromic dye, and the color become visible. At high temperature, the lyochromic dye and the color agent dissolve and disperse in the solvent, and the color disappears. Microcapsules containing solvents with different solidification temperature were used for the detection of different temperature ranges. The humidity sensing units contain cobalt chloride, which is blue in dry state and turns pink when it meets water and forms hydrates. In CO_2_ sensing units, the absorption of CO_2_ forms carbonic acid and changes the color of M‐cresol purple. By tracking the color changes of these sensing units, multimodal signals can be detected and quantified (Figure 1A).

### 2.2 Fabrication, Optimization, and Calibration of the Colorimetric Sensing Units

Five mega‐pixel CMOS imagers (OV5640) with 1.4 µm pixel sizes (Figure S1, Supporting Information) were used to fabricate the M‐imagers. Movie S1 (Supporting Information) shows a noncontact material deposition method, which was applied to fabricate colorimetric sensing units with custom patterns without damaging the delicate microlenses and photodiodes. Porous substrates or sensing materials are usually required to fabricate solid‐state gas sensors to accelerate gas exchanges and increase binding sites.^[^ ^17^ ^]^ To improve the gas sensing performance on the nonporous microlenses, we included silica nanoparticles (SiO_2_ NPs) in the sensing materials for CO_2_ and humidity detection.

**Figure 2:**
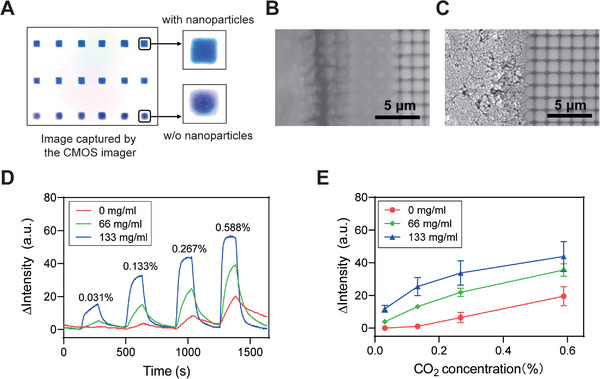
Performance enhancement of the sensing units with nanoparticles, using CO_2_ units as an example. A) Image of sensing units with different concentrations of nanoparticles. B,C) The morphological characterization of sensing units without and with nanoparticles. D) The intensity responses of the sensing units to different gas concentrations over time. E) Comparison of the sensing performance of the sensing units with 0, 66, and 133 mg mL^−1^ SiO_2_ NPs. The error bars represent standard deviations of 6 sensing units in each row of (A).

Taking CO_2_ detection as an example, we fabricated sensing units with different amounts of SiO_2_ NPs on the same imager and compared their performances (**Figure** **2**A). As expected, sensing units with more SiO_2_ NPs demonstrated more porous structures (Figure 2B,C), which drastically increased the surface‐to‐volume ratio of the sensing units. In addition, the light scattering effect of SiO_2_ NPs can prolong the optical path through the matrix of the sensing unit and increase the light absorbance and color change according to Beer–Lambert law. Therefore, the sensitivity was greatly increased and the response time was reduced (Figure 2D,E). However, the addition of too many SiO_2_ NPs may clog the printer nozzle needle. Considering the sensing performance and printability, 133 mg mL^−1^ SiO_2_ NPs were used to fabricate the sensing units.

**Figure 3:**
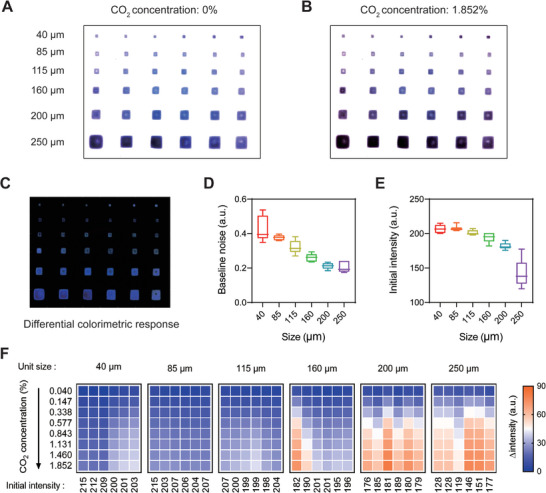
Performance evaluation of sensing units with different sizes and thicknesses, using CO_2_ units as an example. A,B) Images of different size sensing units and their responses to CO_2_ gas. C) shows the differential of (A) and (B). D) Relationship between the unit size and the baseline noise (standard deviation of the intensity level in standard air with 0 ppm CO_2_ over 100 s). E) Relationship between the initial intensity and the unit size in standard air. The error bars represent the minimum/maximum range of the values of 6 sensing units with the same size. F) Intensity responses of the different size sensing units to various CO_2_ concentrations.

To further study the sensitivity and noise dependence of the size of the sensing unit, we printed CO_2_ sensing units ranging from 40 to 250 µm in size on one imager and tested the sensors with CO_2_ ranging from 0% to 1.852% (**Figure** **3**A,B). Differential images showing the color responses of all sensing units were calculated (Figure 3C), and their intensity changes were compared. Large sensing units had low baseline noise levels because more pixels were involved in the digital averaging of the intensity calculation (Figure 3D). When printing each type of sensing unit, the contact angle between the colorimetric ink and the imager surface was held constant; thus, the initial intensity of the sensing unit (relevant to unit thickness) was inverse proportional to its size (Figure 3E). As a result, large sensing units tended to have higher sensitivity than small sensing units (Figure 3F) because the thick sensing material layers increased light absorption according to Beer–Lambert law. However, printing sensing units larger than 200 µm didn't increase the sensitivity due to the excessive blocking of incident light to the CMOS imager. Similar tests were performed on the humidity and temperature sensing units, and comparable conclusions can be drawn according to the results in Figures S2 and S3 (Supporting Information). Therefore, the size of the sensing units can be optimized for different applications according to the integration level and sensing performance requirements.

**Figure 4:**
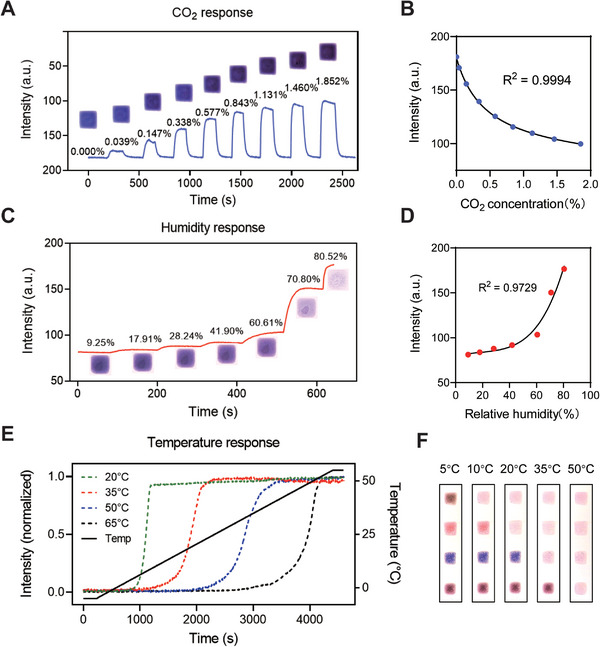
Calibration of the CO_2_, humidity, and temperature sensing units. A) Response of a CO_2_ sensing unit to different concentrations of CO_2_. B) Calibration curve of the CO_2_ sensing unit. The calculated limit of detection (LOD) was 45.98 ppm. C) Response of a humidity sensing unit to different humidity levels. D) Calibration curve of the humidity sensing unit. E) Responses of the 4 temperature sensing units with different transition points during the −5 to 55 °C ramp up. F) Photos of the 4 temperature sensing units at different temperatures.

In this work, 200 µm CO_2_, humidity, and temperature sensing units were used to fabricate the M‐imagers. **Figure** **4** shows the calibration results of these sensing units. For CO_2_ and humidity detection, the sensitivity and dynamic range of a single sensing unit can support environmental monitoring under ambient conditions. For temperature detection, four sensing units containing thermochromic microcapsules with different transition points were used to cover a detection range of 5–50 °C (Figure S4, Supporting Information). Moreover, the parameters of the sensing units can be modified to meet the sensitivity and dynamic range requirements of different applications.

### 2.3 Example Application: Microrobot for Environmental Sensing and Mapping

**Figure 5:**
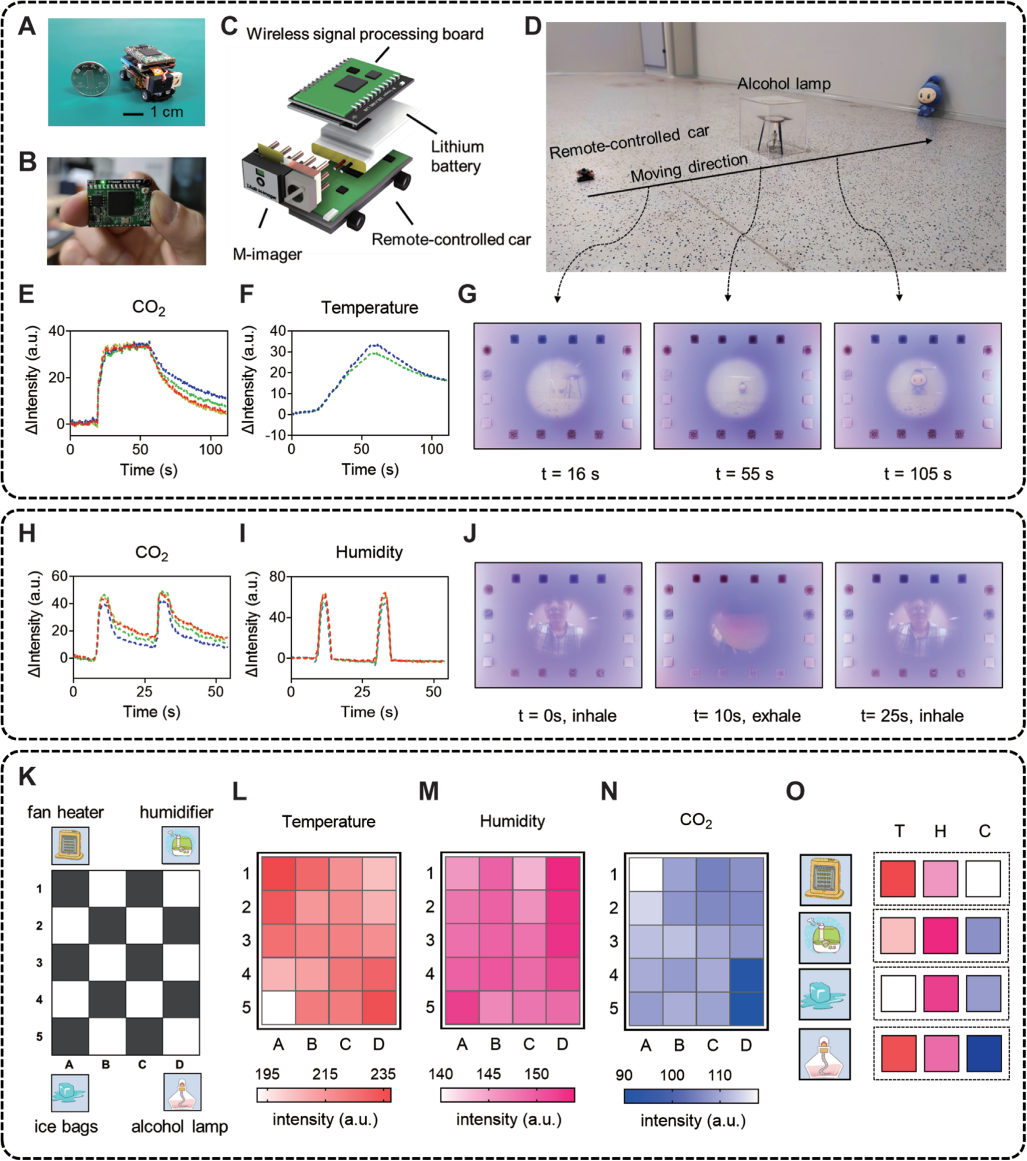
Integration of the M‐imager in a microrobot for environment exploration. A) The remote‐controlled wheeled robot equipped with an M‐imager. B) The wireless data processing module. C) The assembly diagram of the wireless wheeled robot. D) Image of the wheeled robot passing the alcohol lamp. E,F) The responses of the CO_2_ and temperature sensing units during the robot passing the alcohol lamp. The dotted lines in different colors indicate different sensing units. G) Multimodal images captured during the crossing. H,I) The responses of the CO_2_ and humidity sensing units to the breath cycles of a volunteer. J) Multimodal images captured during a breath cycle. K) Multimodal map generation. Components that interfered with the temperature, humidity or CO_2_ distributions were placed at each corner of the rectangle area (200 cm × 160 cm), which was divided into 5 × 4 grids. L–N) The distributions of the temperature, humidity, and CO_2_ in the area. O) Distinct response patterns of the sensing units near the four components.

Microrobots with multimodal sensing capabilities show great potential in various applications, such as environment exploration and disaster rescue in complex terrains. We equipped an M‐imager on a micro remote‐controlled wheeled robot (**Figure** **5**A–C) and tested the environmental sensing and mapping performance of the robot. The speed of the vehicle was maintained at 3 cm s^−1^ during its movement. An alcohol lamp and a stuffed doll were used to simulate a fire and a target to be approached (Figure 5D). Figure 5E,F shows that the M‐imager can detect CO_2_ and temperature changes as the micro vehicle crosses the fire point, and the visual information confirms that the micro‐vehicle gradually approached its target (Figure 5G). In addition to environmental monitoring, the M‐imager can capture the CO_2_ and humidity changes caused by the breathing cycles of a human subject (Figure 5H–J). These results demonstrate that a single M‐imager allows a microrobot to detect potential dangers and vital signs in survivor searching. To verify that the CO_2_ unit is not influenced by humidity changes, we further conducted an atomizing humidifier experiment in Figure S5 (Supporting Information). Videos of these demonstrations are shown in Movies S2–S4 (Supporting Information).

Simultaneous localization and mapping (SLAM) has extensive applications in autonomous vehicle and robot decision‐making.^[^ ^18^ ^]^ Conventional SLAM focuses on spatial information, and including other parameters in environment exploration could greatly enhance environment–robot interactions. We created a 2D space with uneven CO_2_, humidity, and temperature distributions by placing a fan heater, a humidifier, ice bags, and an alcohol lamp at each corner (Figure 5K). The micro‐vehicle traversed the entire space, and maps showing the distributions of the three parameters were constructed (Figure 5L–N). The temperature increased significantly near the fan heater and alcohol lamp, while the CO_2_ level increased only near the alcohol lamp. The humidity declined along the direction of the humidifier spray. The temperature was the lowest near the ice bags, and the humidity increased slightly at this location. In addition to environment mapping, the combinations of these parameters near the 4 objects showed distinct patterns (Figure 5O), indicating that the M‐imager provides more useful information for object recognition than traditional imagers and demonstrating that M‐imager‐equipped robots can better adapt to vision‐constrained environments.

### 2.4 Example Application: Multimodal Endoscope

**Figure 6:**
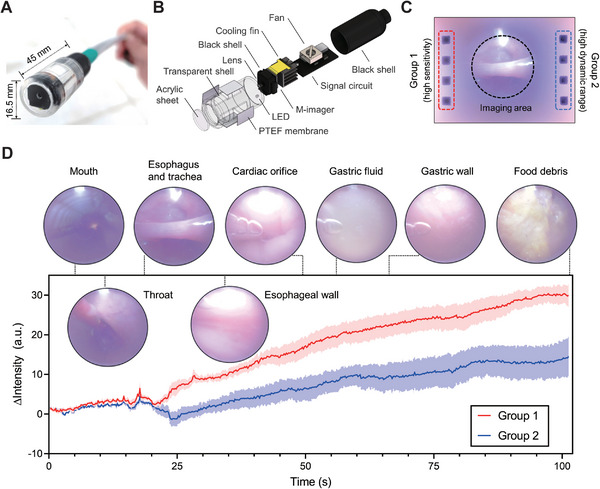
Integration of the M‐imager in endoscopy. A) Photograph of the multimodal endoscopic probe. B) The assembly diagram of the endoscopic probe. C) A multimodal image captured by the endoscopic probe near a Bama pig's esophagus. D) The visual features and responses of the sensing units acquired with the M‐imager module during probe insertion into the digestive tract of the Bama pig. The lines represent the means of the responses of the 4 sensing units of each group, and the undertint areas denote the standard deviations of the 4 sensing units.

Bacterial and tissue metabolism in the human digestive tract produces various gases. Profiling these gases along the digestive tract can provide insights into the functions and health status of the digestive system.^[^ ^10^ ^]^ Conventional endoscopy detects only visual information, and simultaneous detection of chemical and visual information could enable a more comprehensive understanding of the digestive system and more accurate and earlier detection of diseases. However, it has not yet been demonstrated due to the limited space in endoscopes for integrating multiple sensors. To show the superiority and great potential of the M‐imager in medical devices, we built a multimodal endoscope using the M‐imager (**Figure** **6**A,B) and conducted in vivo experiments on a Bama pig (Figure S6, Supporting Information).

As a proof of concept, we detected acid gases in the porcine digestive tract using CO_2_ sensing units, with M‐cresol purple changing color upon exposure to acid gases. To achieve detection with both high sensitivity and a high dynamic range, we coated the original CO_2_ sensing units (Group 1 in Figure 6C) and modified CO_2_ sensing units (Group 2 in Figure 6C) with a high concentration of tetrabutylammonium hydroxide on the same M‐imager. Figure 6D illustrates the visual and chemical information collected along the digestive tract of the anesthetized pig. The results clearly show the visual features of different anatomical sites, such as the mouth, esophagus, and cardiac orifice. In addition, the sensing units revealed the chemical environment in the digestive tract. The small responses from the mouth to the throat showed that the gas composition in the mouth remained relatively constant. The responses increased rapidly from the esophagus to the stomach, most likely due to the concentration gradient of hydrogen chloride that volatilized from the gastric acid. This pilot experiment confirmed that the M‐imager could provide new opportunities in endoscopies. Video of the demonstration is shown in Movies S5 (Supporting Information). Many colorimetric gas sensing recipes are available,^[^ ^17^, ^19^ ^]^ and the multimodal endoscope can be enhanced to detect multiple gases simultaneously by including an array of heterogeneous gas sensing units.

## 3 Discussion

In this work, we proposed a dual‐focus imaging strategy that transforms a CMOS imager into a multimodal sensor. Lens‐based and lens‐free imaging were combined to integrate vision and other sensing modalities on a fingernail‐sized chip. To show the versatility of our approach, we developed an M‐imager that can detect 4 parameters that are involved in the host‐seeking behaviors of mosquitoes, namely, visual features, CO_2_, humidity, and temperature. We also equipped the M‐imager in a micro remote‐controlled vehicle and an endoscopic probe to demonstrate its applicability in robots and medical devices.

In addition to CO_2_, humidity, and temperature sensors, many sensing materials with optical responses have been developed to detect pressure,^[^ ^20^ ^]^ spectra,^[^ ^21^ ^]^ and biochemical substance.^[^ ^22^ ^]^ Thus, the M‐imager can be extended to integrate more sensing modalities. Compared with other multimodal sensors, the M‐imager is more informative because of its visual sensing capability. Furthermore, the vision and other sensing modalities of the M‐imager share the same data format and data interface, thereby preventing complex data processing and fusion procedures. The obtained multimodal images can be easily processed by available tools, including PyTorch, TensorFlow, and other mainstream machine learning frameworks, for tasks such as human–computer interaction^[^ ^23^ ^]^ and pattern recognition.^[^ ^24^ ^]^

CMOS imagers are widely used in every smartphone, tablet, personal computer, and security camera. This increasing demand has promoted the rapid development of CMOS technology, and CMOS imagers as small as 0.575 mm × 0.575 mm have been released (ov6948, OmniVision Technologies). M‐imagers built on these compact imager chips could enable insect‐scale smart robots and ultrathin multimodal endoscopes. In summary, the M‐imager is versatile, compact, and scalable, providing a promising multimodal sensing platform for developing microdevices capable of perceiving complex environments.

## 4 Experimental Section

### Materials

Tetrabutylammonium hydroxide (25% in water), hexadecyltrimethylammonium bromide, m‐cresol purple, glycine, cobalt chloride hexahydrate, isopropanol, ethanol, and ethylene glycol were purchased from Aladdin Inc. Silicon dioxide nanopowder (5–15 nm) was purchased from Sigma‐Aldrich Inc. Thermochromic microcapsules (color‐change temperature: 20, 35, 50, 65 °C; particle size: 2–10 µm) were purchased from Dongfang Color‐change Tech Inc. (Shenzhen, China). Clear resin (Model: RS‐F2‐GPCL‐04) was purchased from Formlabs Inc. All chemical reagents were used directly without further purification. Ultra‐pure water (18 MΩ) was produced by Millipore Direct‐Q.

### Preparation of the Colorimetric Inks

M‐cresol purple (36 mg), glycine (45 mg), and silicon dioxide nanopowder (800 mg) were first dissolved in 6 mL of mixed solution (the volume ratio of water, ethanol, and ethylene glycol was 5:2:5) and shaken for 30 min. Subsequently, 2 mL of tetrabutylammonium hydroxide (25% in water) and 2 mL of hexadecyltrimethylammonium bromide (0.1 mol L^−1^ in ethanol) were added and stirred evenly to obtain a carbon dioxide‐sensitive ink. Cobalt chloride hexahydrate (600 mg) and silicon dioxide nanopowder (300 mg) were dissolved in 2.2 mL of the abovementioned mixed solution and shaken for 30 min to obtain humidity‐sensitive ink. Thermochromic capsules (800 mg) were dissolved in 1 mL isopropanol. Then, 2.5 mL of clear resin was added and stirred evenly to obtain thermosensitive ink. To facilitate Sonoplot printing, the viscosity of the ink should be maintained between 10 and 400 mPa s. The particles in the ink need to be less than 10 µm in size. Meanwhile, the ink needs to be evenly dispersed without precipitation.

### Fabrication of the M‐Imager Chip

Five‐megapixel CMOS imagers (ov5640, OmniVision Technologies) with a 1.4 µm pixel size and 3.7 mm × 2.7 mm imaging area were used to fabricate the M‐imager chips. The power consumption of the imager was about 200 mW at a resolution of 2592 × 1944 pixels and a frame rate of 10 frames s^−1^. An inkjet deposition system (Sonoplot, Microplotter Proto) was employed for the colorimetric ink printing. Before printing, the lens of the CMOS imager was disassembled to expose the imaging area. As shown in Figure S7 (Supporting Information), the colorimetric inks were preserved in a 96‐well plate and aspirated by a nozzle needle. The inks were ejected from the 50 µm nozzle needle by ultrasonic resonance. The printing process is shown in Movie S1 (Supporting Information). First, a square area was patterned on the imaging area under the control of the built‐in software “SonoGuide,” and the square was moistened with colorimetric inks. Second, the nozzle needle was manually moved over the printing area, and a 3–10 V voltage was applied to form a droplet on the wet area. After the solvent evaporated, an M‐imager chip with multiple sensing units was obtained. Ethylene glycol has a lower surface tension and a much slower evaporation rate than water. During the drying process, the concentration of ethylene glycol increases at the edge of the droplet, resulting in a lower surface tension. The scattered nanoparticles flow from the edge to the center along with the liquid, forming a Marangoni flow that compensates for the capillary flow. Thus, the formation of coffee rings was inhibited.

As shown in Figure 1D, a total of 16 sensing units were fabricated on the CMOS imager chip. The upper 4 units were sensitive to CO_2_, and the bottom 4 units were sensitive to humidity. The left and right 4 units with different colors were sensitive to temperature (From top to bottom: black‐65 °C, blue‐50 °C, red‐35 °C, and green‐20 °C).

### Assembly of the M‐Imager Module

Figure S8 (Supporting Information) shows the assembly of the M‐imager module. A sampling fan (UB393‐500) with a size of 9 mm × 9 mm × 3 mm was purchased from SUNON Inc. (Kaohsiung, China, Taiwan). A PCB board was designed with 4 white light LEDs to provide a light source for the sensing unit lensless imaging. The working current of each LED was 12 mA, and the operating voltage was 2.3 V, so the power consumption was 27.6 mW for each LED, and the total power consumption of the M‐imager was about 310 mW. A 3D printed sensor shell was employed to separate the LED and ambient light sources, which achieved dual‐focus imaging on the M‐imager chip surface. Finally, a custom lens with a radius of 2 mm, focal length of 3.6 mm, and viewing angle of 20° was embedded in the shell for visual imaging.

### Optimization and Calibration of the Sensing Units

To evaluate the sensing performance of the colorimetric units, CO_2_, humidity, and temperature calibration apparatuses were manufactured. The structures of these apparatuses are illustrated in Figure S9 (Supporting Information). The MFC controller (ACU10FD‐LC) was purchased from AccuFlow Technology Inc. (Beijing, China). The CO_2_ sensor (SCD30) and humidity sensor (SHT31) were purchased from Sensirion Inc. To calibrate the temperature, a thermoelectric cooler (TEC) component with the Peltier effect was attached to a heat dissipation fan. By applying forward or reverse current on the TEC component, a temperature difference was created to realize cooling or heating. An RTD sensor (Heraeus, PT100) was employed to detect the temperature of the chamber. In addition, CO_2_ (4% CO_2_, 96% N_2_) and standard air (20% O_2_, 80% N_2_) calibration gases were supplied by Jingong Gas Inc. (Hangzhou, China).

To validate the enhancement effect of the nanoparticles on the sensing unit performance, three CO_2_‐sensitive inks with different silica concentrations (0, 66, and 133 mg mL^−1^) were deposited on a CMOS imager, and 6 units were fabricated for each ink (Figure 2A). The CMOS imager was placed in the calibration apparatus. Then, 310, 1330, 2670, and 5880 ppm CO_2_ gases were sequentially flowed into the chamber. The intensity responses of the units were recorded and analyzed.

To evaluate the sensing performance of colorimetric units with different sizes, 6 units with different sizes ranging from 40–250 µm were deposited on a CMOS imager, and 6 units were printed for each size. Then, the CO_2_, humidity, and temperature sensing performances were assessed.

Finally, referring to the assessment of sensing performance of different size units, 200 µm were employed as a typical unit size and calibrated the CO_2_, humidity, and temperature colorimetric responses.

The morphologies of the CMOS imaging area and sensing units were characterized by a scanning electron microscope (Phenom, XL). The optical microscope images were captured by a stereomicroscope (Nikon, SMZ18).

### Demonstrations: Microrobot for Environmental Sensing and Mapping

To validate the applicability of the M‐imager in microrobot applications, a 1.4 cm × 2.2 cm M‐imager data processing PCB was designed. The M‐imager module and data processing board were equipped in a micro (2.0 cm × 4.0 cm) remote‐controlled vehicle. A circuit schematic diagram of the data processing board is shown in Figure S10 (Supporting Information). The CMOS imager was connected to a DSP module via an MIPI interface to convert the digital signal to an image stream. The stream was transmitted to a computer‐ or smartphone‐based terminal through an MCU (MT7268DAN) with an integrated WiFi module. A Li‐ion battery was employed to power the WiFi, DSP, and LED array. Figure 5A–C demonstrates the data processing PCB and the wireless remote‐controlled car equipped with the M‐imager module.

In the proof of concept demonstrations, the micro‐vehicle was controlled to pass the alcohol lamp, monitor the breath cycles, and atomizer. The video stream was transmitted to a smartphone, and the colorimetric responses of the sensing units were recorded. A stuffed doll named “Xiaozhi” (Mascot of Zhejiang Lab) was placed in front of the remote‐controlled car to determine whether the M‐imager could capture visual information.

In another demonstration, 4 components were placed, including a fan heater, humidifier, ice bags and alcohol lamp, at each corner of a black and white checkered blanket. The central area was a rectangle of size 200 cm × 160 cm (Figure S11, Supporting Information). The area was divided into 5 × 4 grids (Figure 5K). The micro‐car was controlled to move around the map in an S‐shape and to stop and take a photo at the center of each grid. The field distributions of the temperature, humidity, and CO_2_ in the area were analyzed. Informed written consent was obtained from the volunteer. Ethical approval was not required, as the experiment was harmless and required no contact.

### Demonstration: Multimodal Endoscope

A Bama miniature pig (male, 40 kg, 12 months) was employed to validate the applicability of the M‐imager in endoscopy. The Bama pig was under quarantine and observation for two weeks before the experiment and was given good care in a separate cage during the quarantine period. After the quarantine period ended, the Bama pig was used for the endoscopic experiment. All experimental procedures were approved by the Laboratory Animal Management and Ethics Committee of Zhejiang Chinese Medical University (animal experiment ethics approval number: IACUC‐20220328‐16).

The M‐imager module in an endoscope was assembled, and the assembly diagram is shown in Figure 6B. The CMOS imager and DSP module were combined in a 3D printed shell. A transparent acrylic sheet and 0603 patch LED were placed on the front of the shell for convenience during CMOS imaging. PTEF membranes (11 mm × 14 mm, IP64) were encircled along the shell to allow air to enter and prevent liquid leaks. A fan was also integrated into the endoscopic probe to accelerate the gas exchange and improve the response speed of the sensing units. In addition, 4 wires connected the DSP module to a computer through a USB protocol.

In total, 8 sensing units were incorporated on the CMOS imager (Figure 6C). The left 4 units (Group 1) used the same formula as the abovementioned carbon dioxide‐sensitive ink. Considering the high concentration of acid gas in the digestive tract of pigs, for the right 4 units (Group 2), the ink formula was modified and 4 mL of tetrabutylammonium hydroxide were added to extend the sensitivity range.

The Bama pig fasted for 12 h before the beginning of the experiment. A 2 mg kg^−1^ dose of propofol was injected to induce anesthesia. After the Bama pig was anesthetized, a breathing tube for oxygen containing 2% isoflurane was inserted into the trachea of the Bama pig. The Bama pig was kept in a prone position to ensure that its esophagus was not compressed (Figure S6, Supporting Information). The endoscope was slowly passed through the pig's mouth into the digestive tract. The video data were instantly transferred and recorded on a computer. The whole experiment lasted 15–20 min, and vital signs (e.g., blood oxygen, breathing, the BIS index) were monitored to confirm the safety and anesthesia state of the Bama pig. After all operations were completed, the Bama pig was euthanized.

## Conflict of Interest

The authors declare no conflict of interest.

## Author Contributions

H.D. and X.Z. contributed equally to this work. D.W., F.Z., and Q.Y. conceived the idea. H.D. designed the device and performed the experiments. X.Z. and Y.C. prepared and optimized the sensing solutions. C.C. assisted with the animal experiments. L.Q. designed the experimental setups. H.D., Y.L., X.C., and Q.L. analyzed the data. D.W., F.Z., Q.Y., W.W., and H.D. wrote the paper and all authors provided feedback.

## Supporting information

Supporting Information

Supplemental Movie 1

Supplemental Movie 2

Supplemental Movie 3

Supplemental Movie 4

Supplemental Movie 5

## Data Availability

The data that support the findings of this study are available in the supplementary material of this article.
