# Supplementary material for: A Multimodal Sensing CMOS Imager Based on Dual‐Focus Imaging
Source: Adv Sci (Weinh). 2023 Mar 2;10(14):2206699. doi: 10.1002/advs.202206699 (PMC10190568; doi:10.1002/advs.202206699)
Supplement: Supplementary file 1 — Supporting Information [file ADVS-10-2206699-s006.pdf]

ADVANCED SCIENCE Open Access

Supporting Information

for *Adv. Sci.*, DOI 10.1002/advs.202206699

A Multimodal Sensing CMOS Imager Based on Dual-Focus Imaging

Hao Dong, Xubin Zheng, Chen Cheng, Libin Qian, Yaoxuan Cui, Weiwei Wu, Qingjun Liu, Xing Chen, Yanli Lu, Qing Yang, Fenni Zhang* and Di Wang**

Supporting Information

A multimodal sensing CMOS imager based on dual-focus imaging

Hao Dong [#], *Xubin Zheng* [#], *Chen Cheng, Libin Qian, Yaoxuan Cui, Weiwei Wu, Qingjun Liu, Xing Chen, Yanli Lu, Qing Yang* [*], *Fenni Zhang* [*], *and Di Wang* [*]

H. Dong, X. Zheng, C. Cheng, L. Qian, Y. Cui, Y. Lu, Q. Yang, D. Wang

Intelligent Perception Research Institute, Zhejiang Lab, Hangzhou, 311100, China.

E-mail: qingyang@zju.edu.cn ; diwang@zhejianglab.com

W. Wu

School of Advanced Materials and Nanotechnology, Interdisciplinary Research Center of Smart Sensors, Xidian University, Shaanxi, 710126, China.

Q. Liu, X. Chen, F. Zhang, D Wang

Biosensor National Special Laboratory, Key Laboratory for Biomedical Engineering of Education Ministry, College of Biomedical Engineering and Instrument Science, Zhejiang University, Hangzhou, 310027, China.

E-mail: fennizhang@zju.edu.cn

Q. Yang

State Key Laboratory of Modern Optical Instrumentation, College of Optical Science and Engineering, Zhejiang University, Joint International Research Laboratory of Photonics, Hangzhou, 310027, China.

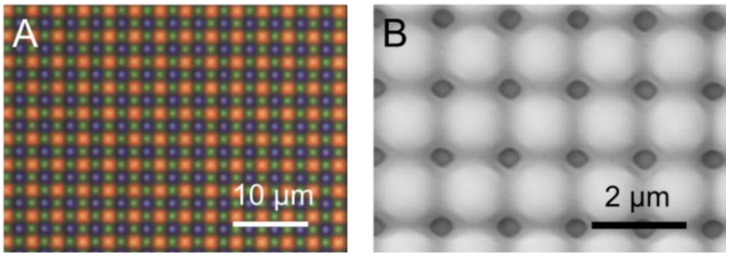

Figure S1. Optical microscope image (A) and SEM image (B) of the OV5640 CMOS sensor.

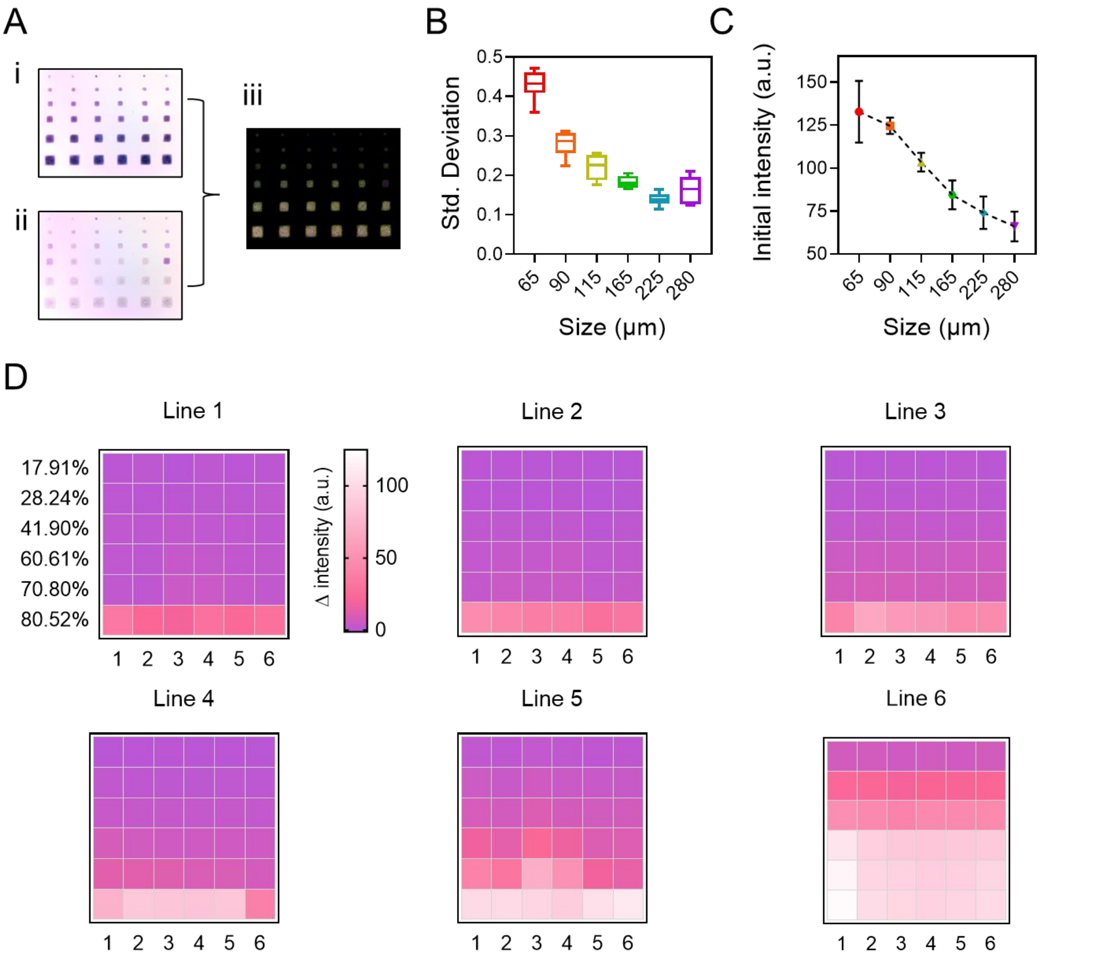

Figure S2. Performance analysis of humidity sensing units with different sizes and thicknesses. (A) Photos captured by an OV5640 CMOS imager with different size sensing units. (i) and (ii) show images acquired by the CMOS imager at RHs of 9.25% and 80.52%, respectively, and (iii) shows the differential of (i) and (ii). (B) Relationship between the intensity std. deviation and the unit size at an RH of 9.25% for 100s. (C) Relationship between the initial intensity and the unit size at an RH of 9.25%. The error bars denote min to max intervals of 6 sensing units with

the same size. (D) Intensity responses of the different size sensing units in environments with various RHs.

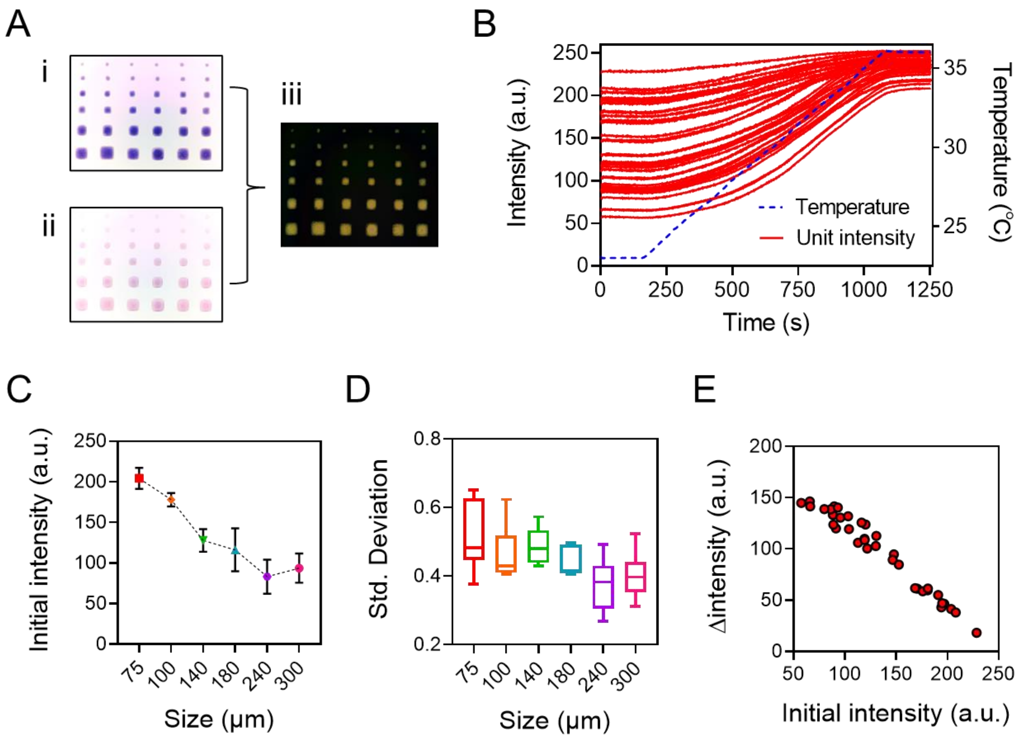

Figure S3. Performance analysis of temperature sensing units (blue) with different sizes and thicknesses. (A) Photos captured by an OV5640 CMOS imager with different size sensing units. (i) and (ii) show the images of the CMOS imager at 23°C and 36°C, and (iii) shows the differential of (i) and (ii). (B) The intensity responses of all the units to the temperature increase from 23°C to 36°C over time. (C) Relationship between the initial intensity and the unit size at 23°C. (D) Relationship between the intensity std. deviation and the unit size at 23°C for 100s. The error bars denote min to max intervals of 6 sensing units with the same size. (E) Relationship between the initial intensity and the intensity responses of the units, which indicates the correlation between the unit thickness and sensitivity.

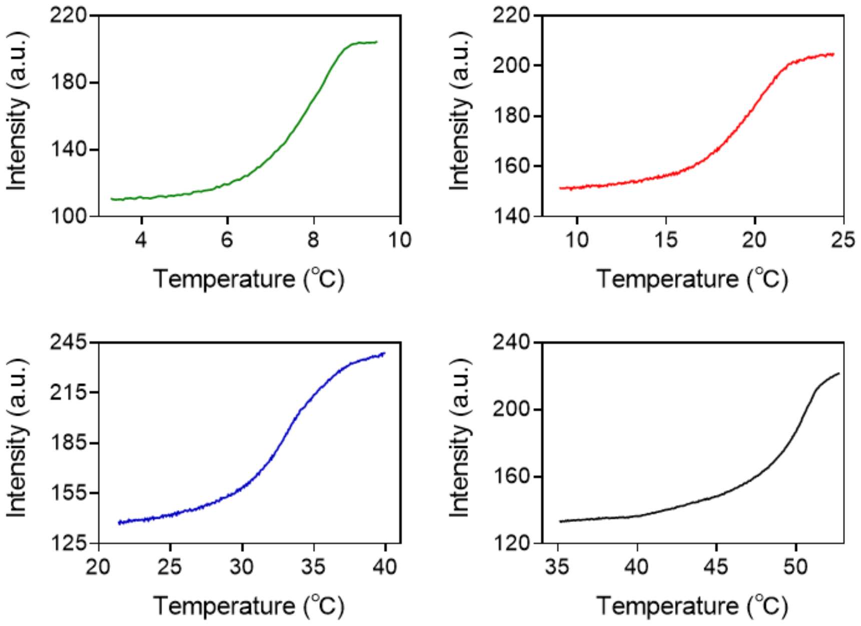

Figure S4. Intensity responses of the four temperature sensing units at different temperatures.

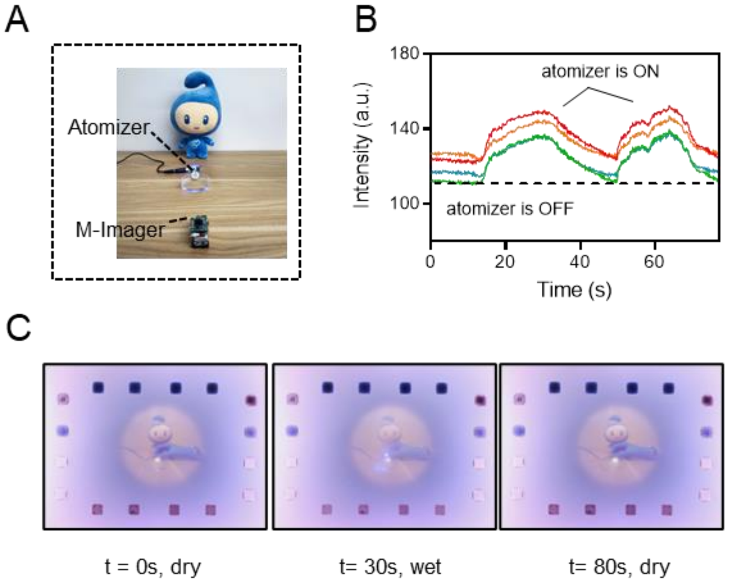

Figure S5. Proof of concept demonstration: atomizer. (A) Photo of the M-imager in front of the atomizer. (B) Intensity responses of the sensing units when the atomizer was on/off. The dotted lines in different colors indicate different sensing units. (C) Photos captured during when the atomizer was on/off.

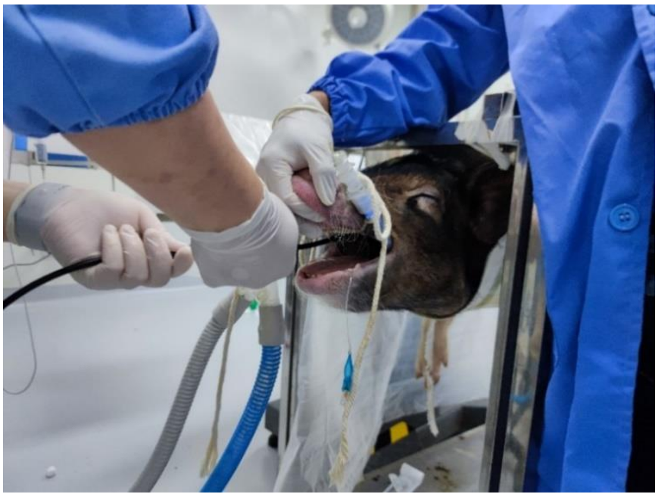

Figure S6. Photograph of the clinical experiment. An endoscopic probe equipped with the M-imager was inserted into the digestive tract of a Bama pig.

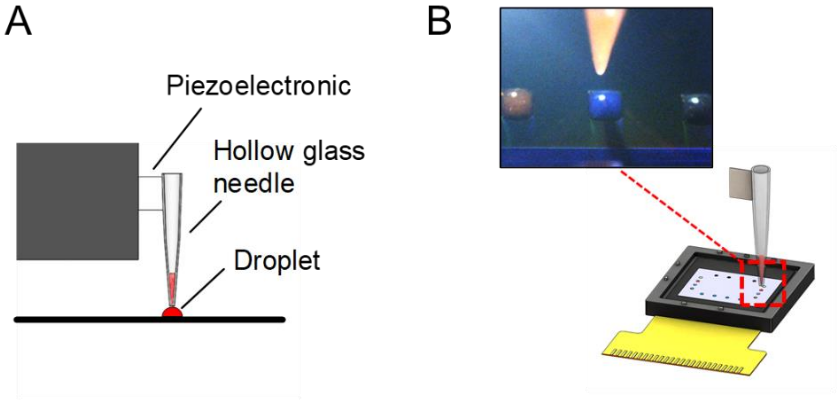

Figure S7. (A) Principle of the inkjet deposition system (Sonoplot, Microplotter Proto). By applying a voltage to the piezoceramics, ultrasonic resonance is induced, which causes the colorimetric ink to be ejected from the needle. (B) Photograph of the deposition procedure for temperature sensing units.

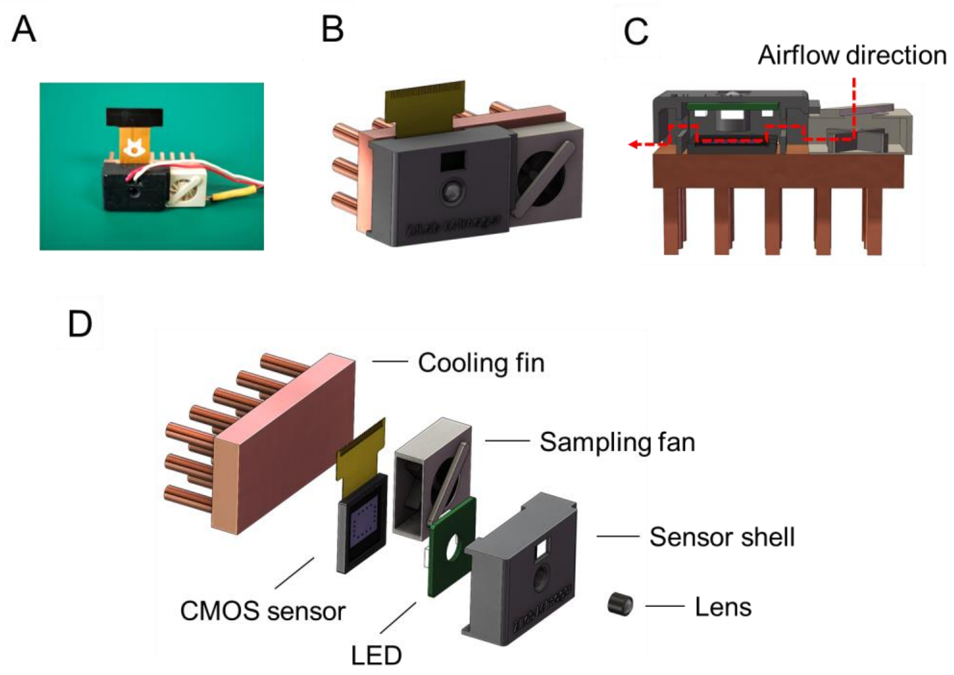

Figure S8. The structure of the M-imager module. (A) Photograph and (B) rendering of the module. (C) The airflow direction inside the module. Ambient gases are purged into the CMOS chamber by a sampling fan. (D) The assembly diagram of the module.

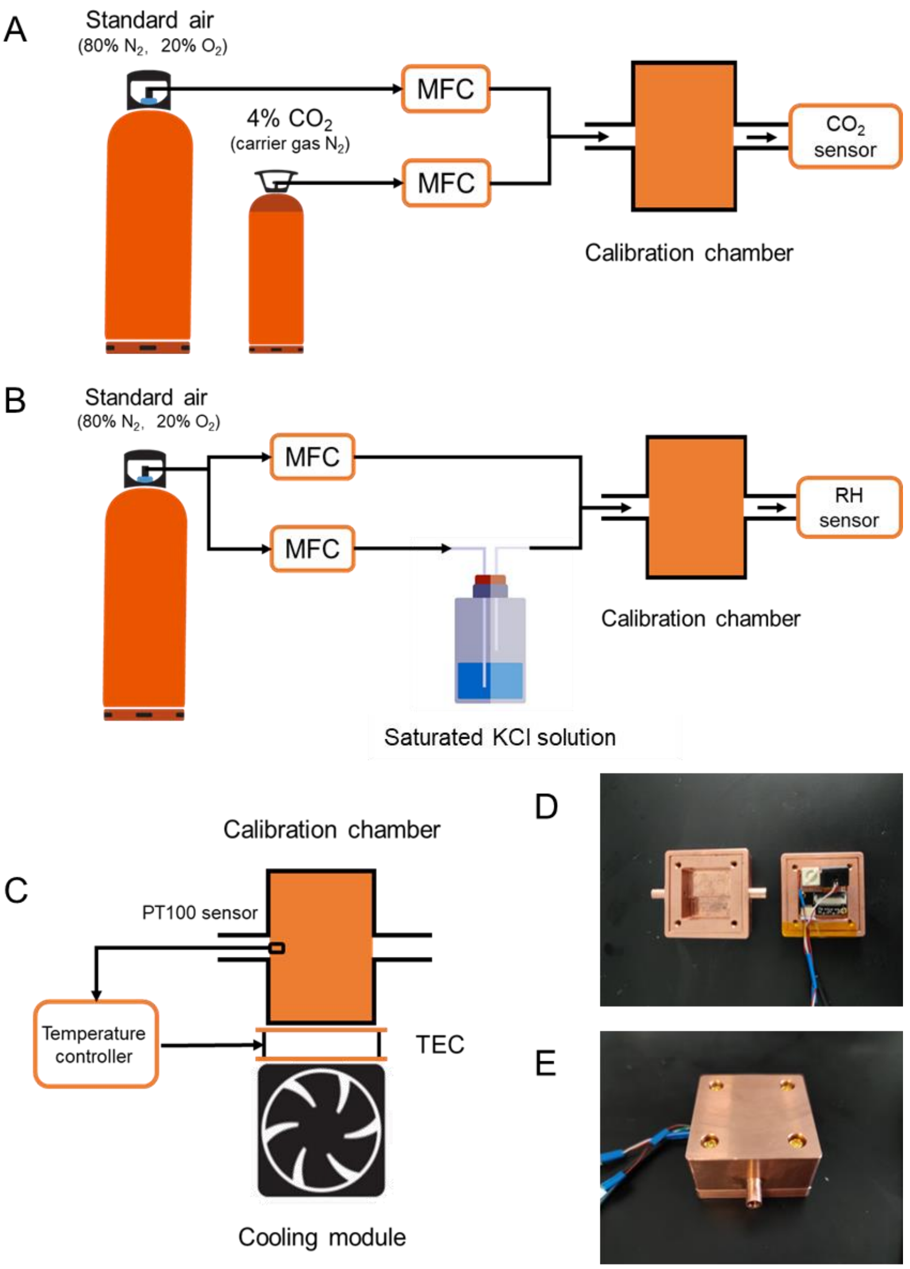

Figure S9. Structures of the calibration apparatuses. (A) CO_2 calibration apparatus. The sample gases are generated by mixing standard air and 4% CO_2 gas and are flowed into the calibration chamber and commercial sensor. The M-imager is placed inside the chamber. (B) Humidity calibration apparatus. Standard air is moistened by a saturated KCl solution, and the relative humidity (RH) is controlled by adjusting the ratio of the moisture gases. (C) Temperature calibration apparatus. A thermoelectric cooler (TEC) component with the Peltier effect is employed as the power element to heat and cool the chamber. (D) and (E) Photographs of the calibration chamber.

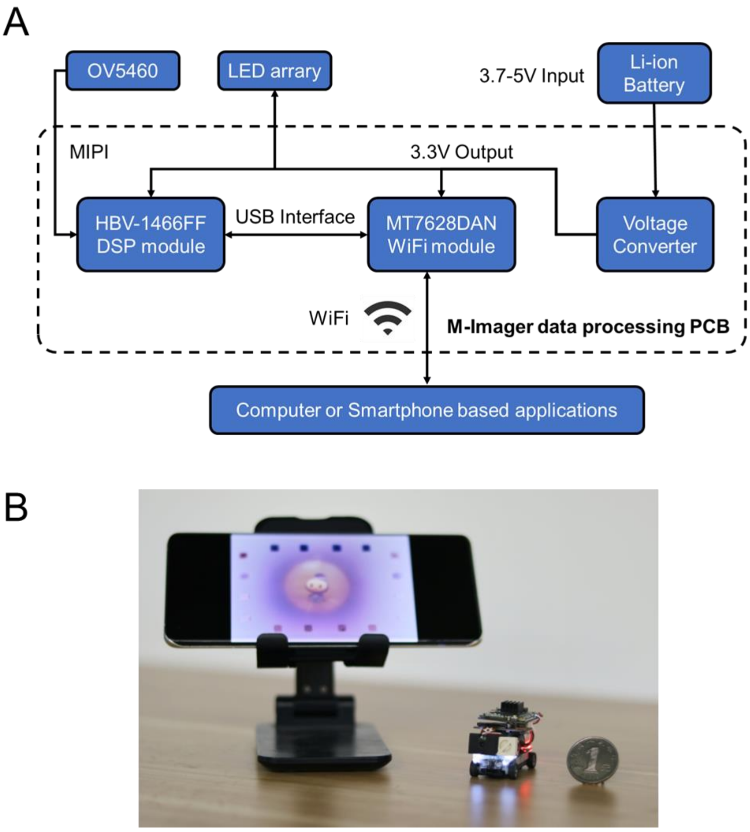

Figure S10. Diagram of the M-imager data processing module. (A) The circuit schematic of the data processing PCB for the M-imager. (B) Photograph illustrating the signal transmission from the M-imager to a smartphone.

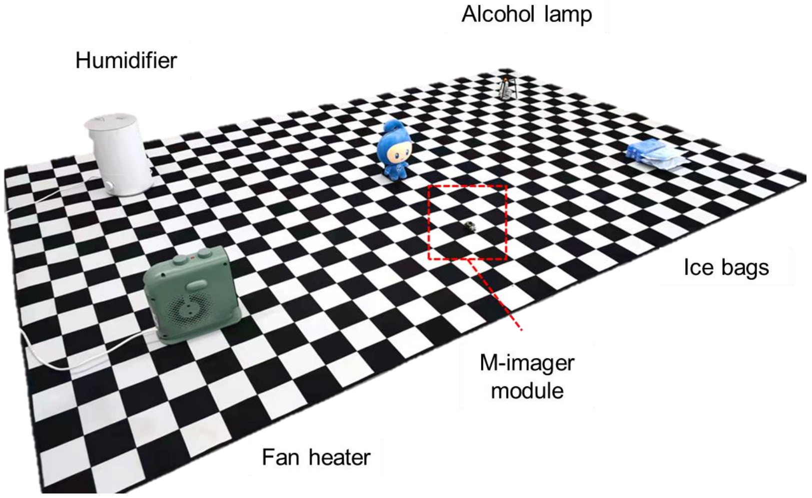

Figure S11. Layout of the map with interference factors. Components that interfere with the temperature, humidity and CO_2 distributions in the ambient environment were placed on each side of the map, and the central area was a 200 cm × 160 cm rectangle.

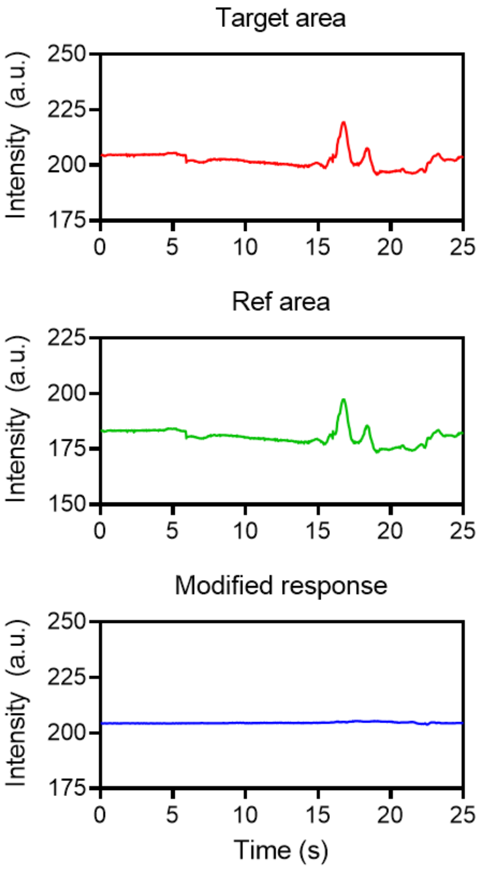

Figure S12. The compensation effect of Equation (1) in reducing ambient light interference.

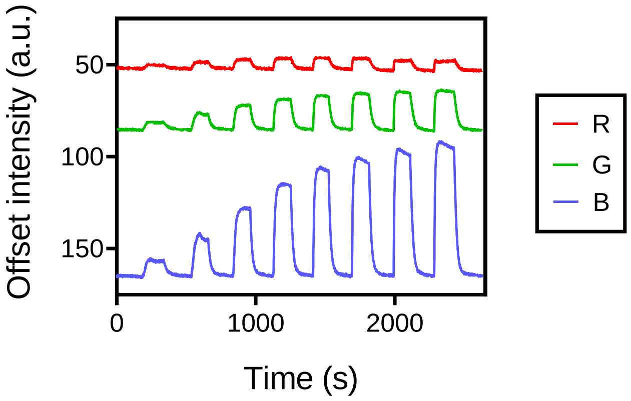

Figure S13. Typical intensity responses of a CO_2 sensing unit to different gas concentrations. The R, G, and B channels were divided, and the B channel showed the best sensitivity.

Data analysis

The video stream was transmitted to a smartphone in AVI format at a resolution of 2592×1944 pixels and frame rate of 10 frames/s. We converted the video file to an image sequence in JPG format using the software "Free video to JPG converter". Then, we imported the image sequence to the open source software ImageJ and split the images into gray and R, G, and B channel intensity stacks. The area of each sensing unit was input, and the mean intensity of the pixels in the area was calculated. We used the mean intensity stacks to quantitatively characterize the color change in the sensing units over time. For the endoscope application, a video resolution of 1600×1200 pixels and a frame rate of 10 frames/s were utilized to ensure that the CMOS chip did not overheat in vivo.

To reduce background color interference, an adjacent area with the same shape and size was selected as a reference. The response of the sensing unit was modified as follows:

$$I(t) = I_{unit}(t) - I_{ref}(t_0) + I_{ref}(t) \quad (1)$$

where $I_{unit}(t)$ and $I_{ref}(t)$ represent the mean intensity of the pixels in the sensing unit and reference region at time t, respectively, and $I_{ref}(t_0)$ represents the mean intensity of the pixels in the reference region at the beginning of the measurement.

The compensation effect of Equation (1) is shown in Figure S12. We placed an M-imager in an environment with fluctuated illumination, and the intensity changes of two adjacent imaging areas were calculated, one as a target signal and the other as a reference signal. The compensated signal obtained by Equation (1) eliminated most of the noise caused by ambient light fluctuations.

Figure S13 demonstrates a typical intensity response of a CO_2 sensing unit. The unit color changed from blue to yellow, and the intensity responses of the R, G, and B stacks showed different sensitivities. In this circumstance, we employed the most sensitive channel (Blue) to represent the CO_2 concentration. The same evaluation method was applied to select the blue channel for the 20°C temperature unit, CO_2 unit and humidity unit. The green channel was chosen for the 35°C temperature unit. The

red channel was chosen for the 50°C temperature unit. The gray channel was chosen for the 65°C temperature unit.

In the CO_2 calibration, we employed a binding-saturation equation to fit the correlation between the intensity responses and gas concentrations. The equation is defined as:

$$C_{CO2} = B \times \frac{i_{co2}}{K + i_{co2}} + N * i_{co2} + X \quad (2)$$

where C_{CO2} represents the concentration of the gas samples and i_{co2} represents the intensity response of the sensing unit. B, K, N, and X are parameters in the binding-saturation equation.

To calculate the detection limit, we kept the sensing unit in standard air (0 ppm CO_2) for 100 s. The $Signal_{threshold}$ was defined based on the zero concentration responses (mean: $Signal_{zero}$, standard deviation: Std_{zero}) with the following equation:

$$Signal_{threshold} = Signal_{zero} - 3 \times Std_{zero} \quad (3)$$

The detection limit was obtained by substituting $Signal_{threshold}$ into the calibration curve fitted according to Equation (2).

In the humidity calibration, we employed a power series equation to fit the correlation between the intensity responses and relative humidity. The equation is defined as:

$$RH = A \times {i_{rh}}^{B} + C \times {i_{rh}}^{D} \quad (4)$$

where RH denotes the relative humidity and i_{rh} represents the intensity response of the sensing unit. A, B, C, and D are parameters in the power series equation.

In the temperature calibration, the intensity responses were normalized to compare the responses of the 4 sensing units. The normalization was formulated as follows:

$$I_{normalized}(t) = \frac{I(t) - I_{min}}{I_{max} - I_{min}} \quad (5)$$

where $I(t)$ represents the intensity response with background correction at time t, and I_{min} and I_{max} represent the minimum and maximum response values of the sensing unit during the calibration.
